# An OMOP-CDM based pharmacovigilance data-processing pipeline (PDP) providing active surveillance for ADR signal detection from real-world data sources

**DOI:** 10.1186/s12911-021-01520-y

**Published:** 2021-05-17

**Authors:** Hyunah Shin, Suehyun Lee

**Affiliations:** 1 Health Care Data Science Center, Konyang University Hospital, 158 Gwanjeo-dong-ro, Seo-gu, Daejeon, Republic of Korea; 2 Departments of Biomedical Informatics, Konyang University College of Medicine, 158 Gwanjeo-dong-ro, Seo-gu, Daejeon, Republic of Korea

**Keywords:** Pharmacovigilance, EHR, FAERS, CDM, ADRs, PTA

## Abstract

**Background:**

Adverse drug reactions (ADRs) are regarded as a major cause of death and a major contributor to public health costs. For the active surveillance of drug safety, the use of real-world data and real-world evidence as part of the overall pharmacovigilance process is important. In this regard, many studies apply the data-driven approaches to support pharmacovigilance. We developed a pharmacovigilance data-processing pipeline (PDP) that utilized electronic health records (EHR) and spontaneous reporting system (SRS) data to explore pharmacovigilance signals.

**Methods:**

To this end, we integrated two medical data sources: Konyang University Hospital (KYUH) EHR and the United States Food and Drug Administration (FDA) Adverse Event Reporting System (FAERS). As part of the presented PDP, we converted EHR data on the Observation Medical Outcomes Partnership (OMOP) data model. To evaluate the ability of using the proposed PDP for pharmacovigilance purposes, we performed a statistical validation using drugs that induce ear disorders.

**Results:**

To validate the presented PDP, we extracted six drugs from the EHR that were significantly involved in ADRs causing ear disorders: nortriptyline, (hazard ratio [HR] 8.06, 95% CI 2.41–26.91); metoclopramide (HR 3.35, 95% CI 3.01–3.74); doxycycline (HR 1.73, 95% CI 1.14–2.62); digoxin (HR 1.60, 95% CI 1.08–2.38); acetaminophen (HR 1.59, 95% CI 1.47–1.72); and sucralfate (HR 1.21, 95% CI 1.06–1.38). In FAERS, the strongest associations were found for nortriptyline (reporting odds ratio [ROR] 1.94, 95% CI 1.73–2.16), sucralfate (ROR 1.22, 95% CI 1.01–1.45), doxycycline (ROR 1.30, 95% CI 1.20–1.40), and hydroxyzine (ROR 1.17, 95% CI 1.06–1.29). We confirmed the results in a meta-analysis using random and fixed models for doxycycline, hydroxyzine, metoclopramide, nortriptyline, and sucralfate.

**Conclusions:**

The proposed PDP could support active surveillance and the strengthening of potential ADR signals via real-world data sources. In addition, the PDP was able to generate real-world evidence for drug safety.

**Supplementary Information:**

The online version contains supplementary material available at 10.1186/s12911-021-01520-y.

## Background

Adverse drug reactions (ADRs) are among the top 10 leading causes of death and result in annual costs of approximately $75 billion in the United States (US); hence, they represent a significant public health concern [1, 2]. Currently, two major methods have been developed for pharmacovigilance: premarketing review and post-marketing surveillance [3]. As it is impossible to test every possible interaction between a new drug and existing drugs using in vivo or in vitro assays in a premarketing review, post-marketing surveillance for the detection of potential ADRs is widely identified as a necessity.

Post-marketing surveillance typically engages a number of approaches to monitor drugs, using real-world data [4]. Real-world data usually includes information such as the spontaneous reporting system (SRS), electronic health records (EHR), and medical claims data. Real-world evidence typically refers to clinical evidence regarding the usage and potential benefits or risks of a medical product derived from analysis of real-world data [5]. Of these data sources, SRS data is a critical resource for the detection of ADRs because it contains data collected directly from patients and healthcare professionals. However, as pharmacovigilance relying on only one data source has limitations, an approach combining different data sources should be considered. Recent studies have used clinical center or hospital EHR data, the United States Food and Drug Administration (FDA) Adverse Event Reporting System (FAERS) data, claims data and social media, etc. A study showed that EHR data can be used in a complementary manner to improve signal detection from FAERS [6].

Widely accepted standardized data structures are also referred to as common data models (CDM) and they are used to efficiently utilize data from hospitals. The use of a CDM is beneficial as it unifies the terms used in each medical institution into standard terminology. Different types of CDM are used according to the purpose of the study, such as the Sentinel CDM [7] and the Observation Medical Outcomes Partnership (OMOP) CDM from the Observational Health Sciences and Informatics (OHDSI) [8]. OHDSI is a public–private partnership supporting clinical effectiveness research, and aims to develop tools for the analysis of observational data and open source research resources for collaborative research. There are many studies emphasizing on the use of OMOP-CDM for signal detection [9–12]. The development of CDM has evolved to converge various real-world data, however, CDM is still used based and focused on EHR data, and there are some limitations. In particular, for pharmacovigilance, since drugs and ADRs terms used by clinical institutions are different, and variables depend on study design or objectives, appropriate data processing is in needed.

In this study, we used two different real-world data sources: EHR and SRS data, and developed a pharmacovigilance data-processing pipeline (PDP) that converts the two data sources to OMOP CDM. The process includes standardizing terminology, transforming and loading each EHR and SRS data tables into OMOP CDM. We devised the objectives of PDP to further validate detected novel drug-ADR signals with different real-world data sources, and especially using laboratory and measurement results from EHR data. We used the laboratory and measurement results as supplementary data for statistical analysis and conducted meta-analysis on the signals, we could identify the consensus results from heterogeneous data.

We extracted pure tone audiometry (PTA) reports from the EHR data for the detection of ADRs resulting in ear disorders which we chose as the target ADR to evaluate the PDP. Hearing loss is one of the most chronic disabilities and the World Health Organization (WHO) has estimated the annual cost of unaddressed hearing loss to be approximately $750 billion [13, 14]. Although it is a preventable disease, it is difficult to cure once it has occurred; thus, early diagnosis is necessary. PTA is a test that measures the patient’s hearing threshold compared with the average normal threshold at various frequencies. Typically, PTA reports are used to analyze the relationship between certain diseases and hearing loss, or to determine the status of hearing loss in patients including pharmacovigilance (PV) studies [15–17].

## Methods

### Pharmacovigilance data-processing pipeline (PDP)

For systematic pharmacovigilance, we developed the specialized data process pipeline for data cleansing (e.g., conversion of clinical data which is collected graph image-based such as PTA reports) related to drugs and ADRs from different real-world data sources. Since it is difficult to detect and assess the drug-induced ADRs from only one data source, we converted EHR and FAERS data into OMOP CDM for integrating actual ADR reports and clinical data. Yu, Yue, et al. study [18] proposed the newly pharmacovigilance platform that converting FAERS data to OMOP CDM and detecting signals from the platform. Compared to the previous study [18], the presented PDP converts unstructured data from EHR laboratory and measurement results relevant to ADR symptoms to structured data. EHR includes data that are clearly standardized in codes or numerical values, such as drug prescription, diagnosis, or laboratory test (blood, urine, etc.) and are obtained based on signals or graph image, such as polysomnography, electrocardiography, and audiometry test (pure-tone, speech, etc.). Specifically, we used the structured data to supplement information and detected potential ADR signals for OMOP CDM based pharmacovigilance.

**Fig. 1:**
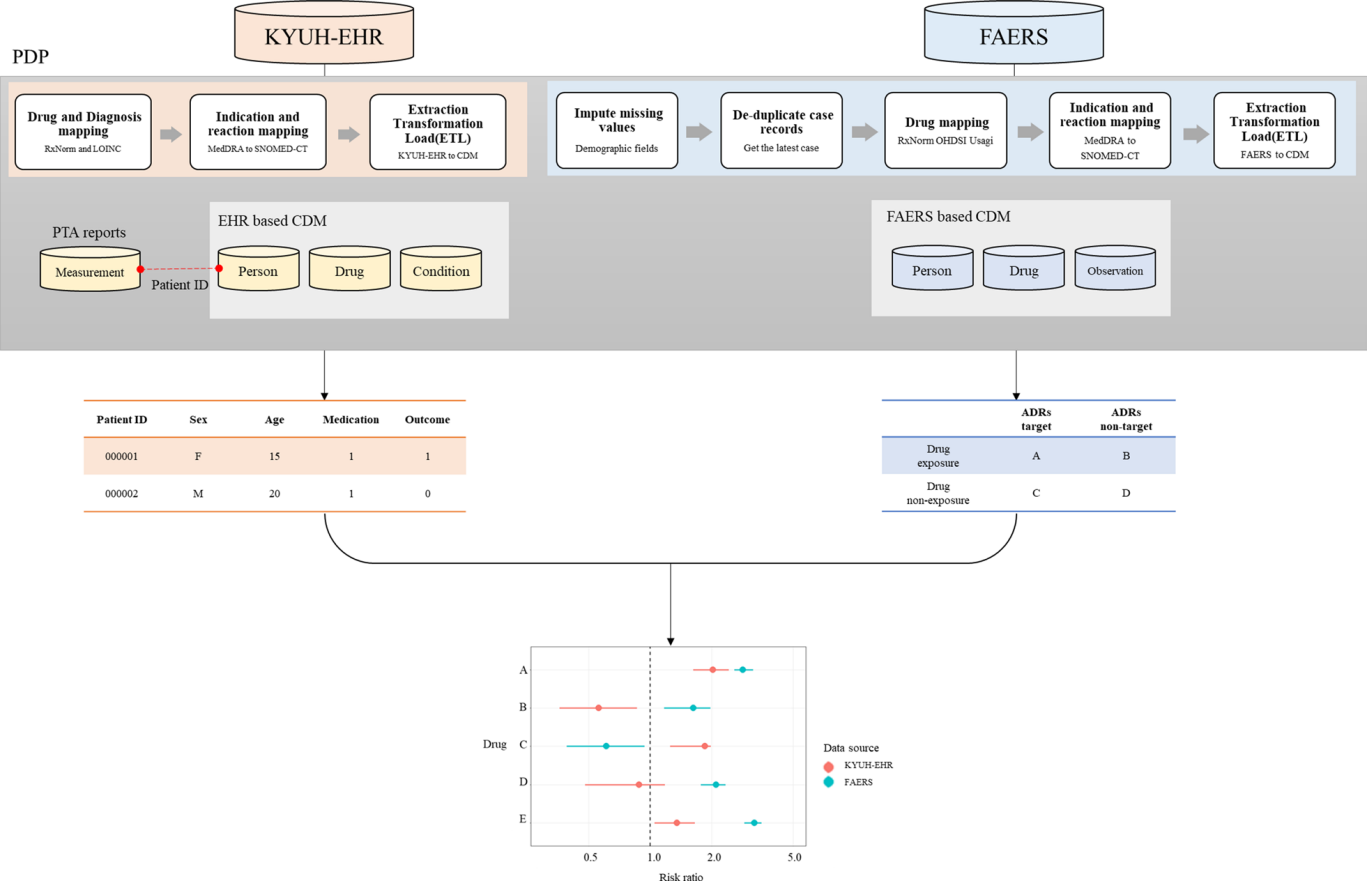
Overview of PDP for active surveillance of ADRs from real-world data sources

The representative unstructured data used in this study, PTA reports, results in a graph image. Therefore, we manually input numerical values into CDM table, and categorized the condition for normal or abnormal based on the values. Similar to PTA reports, various case report form (CRF) data are missed or could not be inserted to the OMOP CDM tables. Since there are different reports data that can be used for identifying disorders of patients, it is important to design study accordingly. Using the appropriate clinical test indicators, we can identify whether a drug induced ADRs based on evidence. The overview of PDP used for active surveillance of drug-ADRs is shown in Fig. 1.

#### EHR

We analyzed all EHR data for patients obtained from January 1, 2014 to December 31, 2018 of Konyang University Hospital (KYUH), comprising a total of 347,040 patients. Each EHR contains information on admissions, discharges, drug prescriptions, laboratory results, and nursing documents. As the EHR data includes a patient’s personal information, a new patient ID was assigned to prevent re-identification when the data were converted with the PDP. Diagnosis information, visit information, and prescription information corresponding to the newly assigned patient ID were mapped and could be visualized for each patient. The PTA reports of information contained in the EHR tests table was confirmed by connecting the patient ID with the primary key in the person table.

#### FAERS

FAERS contains seven tables, for patient demographic and administrative information (DEMO), drug/biologic information (DRUG), adverse events (REAC), patient outcomes (OUTC), report sources (RPSR), drug therapy start and end dates (THER), and indications for use/diagnosis (INDI) [19]. The raw data from FAERS, from 2012 to 2018, were preprocessed to remove duplicate cases, replace missing values, and map drug names to RxNorm. The preprocessing was conducted using Adverse Event Open Learning Through Universal Standardization (AEOLUS) developed by Banda et al., [20]. Standardization was performed first in PDP and, as shown in Fig. 1, the tables used for analysis were person, drug, and observation.

### PTA reports

**Fig. 2:**
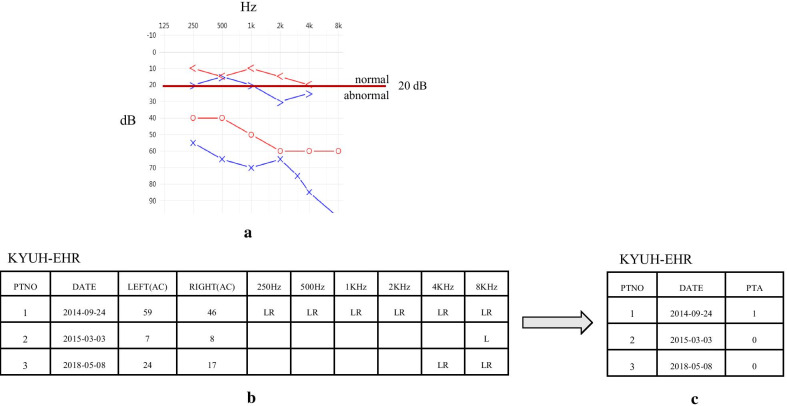
Extraction and conversion of data from PTA results (AC: Air conduction Audiometry, LR: abnormal in Left & Right, L: abnormal in Left)

PTA reports were related to EHR data by patient ID, and the data were stored as an image file containing a results graph (Fig. 2a). Therefore, we individually inspected the image files and manually recorded the pure tone threshold average6 (PTA6) for the left and right ear, for approximately 7800 patients. In addition, we recorded the abnormal ear values for each frequency (Fig. 2b). We determined the patient’s state as abnormal when values for each frequency were above 20 dB [21]. Finally, for each patient, we converted normal or abnormal status for the PTA to categorical data in the EHR (Fig. 2c).

### Drug selection

**Fig. 3:**
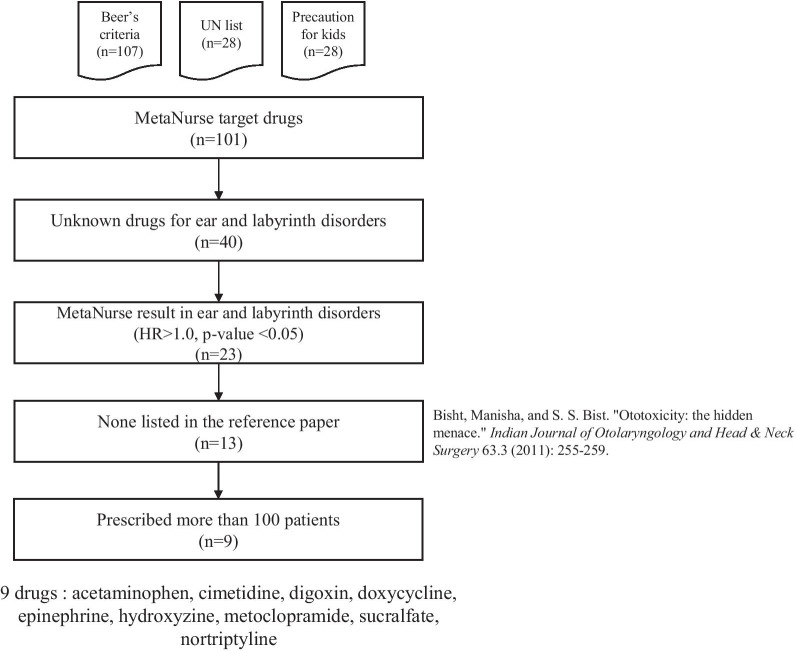
Process for target drug selection

We selected drugs based on the activities–enabled pharmacovigilance MetaNurse algorithm [22], which includes Beers criteria medications (n = 107) [23, 24], medications with precautions for kids (n = 79) [25–27], and the United Nations’ marketing prohibition drug list (n = 28) [28, 29]. Of the 101 drugs that were final targets of MetaNurse, 23 drugs that were significantly associated with ear and labyrinth disorders were selected; that is, the HR was greater than 1.0, and the p-value was less than 0.05. In conclusion, 9 drugs that were not listed in the ototoxicity definition paper [30] but were prescribed to more than 100 patients were selected as the potential drugs of interest for the current study, as shown in Fig. 3.

### Outcome definition

**Fig. 4:**
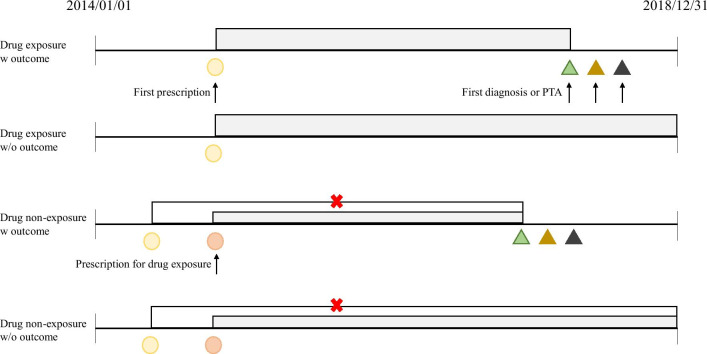
Time definition for the drug-exposure and non-exposure group in Cox analysis

We applied 1:1 propensity score matching (PSM) to each of the 13 tables and then performed Cox proportional hazard regression analyses to calculate the HR and 95% confidence interval. Propensity score matching and Cox proportional hazard regression analyses were computed. The independent variables were gender, age, number of visits (only those subject to outpatient care), and hospitalization period (only those subject to inpatient care), and the dependent variable was set as taking or not taking the target drug. We matched the date of the drug prescription for patients who were not taking the target drug to the date of prescription for patients who were taking the target drug. The above process is shown in Fig. 4. A time variable is required to proceed with the Cox analysis. In determining the time, we set the period between the date of the prescription and the date of the first diagnosis with ear disorders or abnormal PTA test, or if there was no diagnosis, it was set to the period from the date of the drug prescription to December 31, 2018.

### Statistical analysis

In EHR, we created an analysis table based on drug and condition tables, each including prescription and diagnosis information. When the patient was prescribed a target drug, it was categorized as 1 for Medication in each table. When diagnosis of ear disorders and abnormal PTA test occurred within 365 days after the prescription of the target drugs, this was categorized as 1 for Outcome. Then, we processed the diagnosis and PTA information independently.

In FAERS, with each reported case counted as one case, the number of drug and ADRs pairs was created using a 2 × 2 contingency table [31]. **A** indicates the number of co-occurrences of interest. The number of co-occurrences with a drug of interest but without an ADR of interest, was defined as **B**, and the number of co-occurrences without a drug of interest but with an ADR of interest was defined as **C**. **D** indicates the number of co-occurrences without either drug or ADR of interest. We calculated the odds ratio between the two variables, and determined whether it was a significant outcome by considering the 95% confidence interval and the p-value. Finally, we performed a meta-analysis using Forest plot in R. This method was developed for use in medical research as graphical representation of a meta-analysis of the results of randomized controlled trials [32].

## Results

### PDP statistics

As the two data sources corresponded to SRS and EHR data, respectively, we were able to examine different aspects of the data. The characteristics of each data source are shown in Table 1. The total number of reports was 6,950,486 in the FAERS reports, and EHR data included 347,040 patients. For drugs, there were 24,609,422 prescription records in the EHR and more than the 24,022,269 drug administrations were reported in FAERS reports. EHR was analyzed using a condition occurrence table containing 8,095,757 diagnoses, and FAERS was analyzed using an observation table containing 26,366,828 ADRs.

**Table 1:** Characteristics of KYUH-EHR and FAERS

	KYUH-EHR	FAERS
Years	2014–2018	2012–2018
Patients Reports	347,040 –	– 6,950,486
Sex		
Male Female Unknown	169,197 (48.8%) 177,843 (51.2%) –	2,385,735 (34.3%) 3,829,814 (55.1%) 734,937 (10.6%)
Drug_exposure	24,609,422	24,022,269
Condition_occurrence	8,095,757	–
Observation	–	26,366,828

### Case definition

**Fig. 5:**
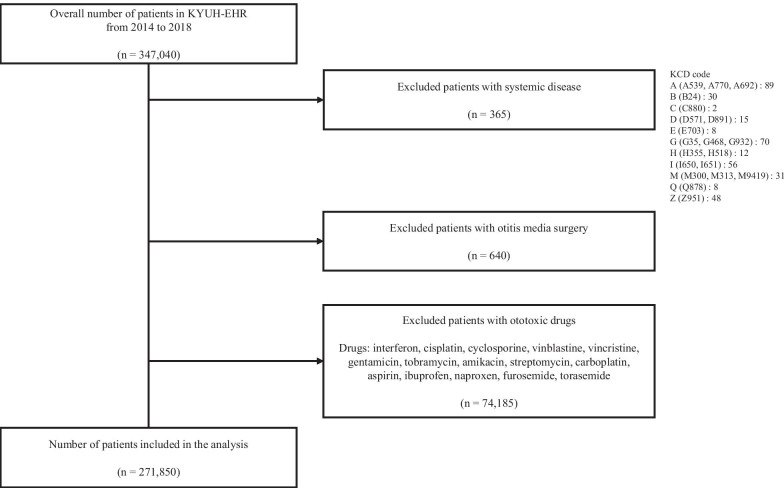
Case definition and criteria in KYUH-EHR

The case definition and the selection criteria used to select patients in EHR are shown in Fig. 5. We excluded patients with systemic diseases from a total of 347,040 patients who visited KYUH from 2014 to 2018. Because this is a study detecting adverse drug event-inducing drugs, we excluded patients who had systemic diseases that cause ear disorders in order to reduce the effect from different variables. In consultation with an otolaryngologist, we selected multiple sclerosis, albinism, lyme disease, cryoglobulinaemia, polyarteritis nodosa, wegener’s granulomatosis, multiple sclerosis and etc., as systemic diseases, which excluded 365 patients. The codes associated with systemic diseases can be found in Fig. 5. Then, we excluded a further 640 patients who had undergone otitis media surgery, and 74,185 patients who were prescribed drugs known to be for ear disorders (e.g., interferon, cisplatin, cyclosporine, vinblastine).

### Analysis of results from KYUH-EHR and FAERS

We found that six drugs, acetaminophen, digoxin, doxycycline, metoclopramide, nortriptyline and sucralfate, had significant signals. Nortriptyline had the highest HR, at 8.06, followed by metoclopramide (HR, 3.35), doxycycline (HR, 1.73), digoxin (HR, 1.60), acetaminophen (HR, 1.59), and sucralfate (HR, 1.21). Of the 255 people who took nortriptyline, 23 (9.01%) were found to have ear disorders; in contrast, ADRs were detected in 4 (1.57%) of the 255 patients who did not take nortriptyline. Of the 255 patients who took nortriptyline, 85 were men (33.33%), and 170 were women (66.66%). The average age was 51.4 ± 16.6 years for men, 52.4 ± 15.2 for women, the average number of outpatient visits was 9.7 ± 9.5 for men, 10.0 ± 7.9 for women, the average length of hospitalization was 2.9 ± 7.8 days for men, and 1.4 ± 3.0 for women.

In the FAERS data, four drugs, doxycycline, hydroxyzine, sucralfate, and nortriptyline, were found to have significant signals. FAERS also had the highest signal for nortriptyline (ROR, 1.94), similar to EHR, followed by sucralfate (ROR, 1.22), doxycycline (ROR, 1.30), and hydroxyzine (ROR, 1.17). Of the 8,052 cases taking nortriptyline, 343 (4.3%) cases were considered to have ear disorders; the incidence was higher in women (271 [79%] cases) than in men (56 [16.3%] cases), with sex not specified in 16 (4.7%) cases (Table 2).

**Table 2:** Characteristics of each analysis and signals for drug

Study	KYUH-EHR	FAERS
Area (year of enrollment)	Korea (2014–2018)	U.S.A (2012–2018)
Adjustment	Sex, age, hospitalization period, number of outpatient visits	–
Drug	Hazard ratio (95% CI)	Odds ratio (95% CI)
Acetaminophen	1.59 (1.47–1.72)*	0.80 (0.77–0.84)
Cimetidine	0.74 (0.61–0.91)	0.98 (0.69–1.36)
Digoxin	1.60 (1.08–2.38)*	0.73 (0.66–0.80)
Doxycycline	1.73 (1.14–2.62)*	1.30 (1.20–1.40)*
Epinephrine	1.03 (0.91–1.18)	0.62 (0.53–0.72)
Hydroxyzine	0.98 (0.72–1.34)	1.17 (1.06–1.29)*
Metoclopramide	3.35 (3.01–3.74)*	0.80 (0.72–0.90)
Nortriptyline	8.06 (2.41–26.91)*	1.94 (1.73–2.16)*
Sucralfate	1.21 (1.06–1.38)*	1.22 (1.01–1.45)*

^*^^P^^<^^0.05^

### Meta-analysis

**Fig. 6:**
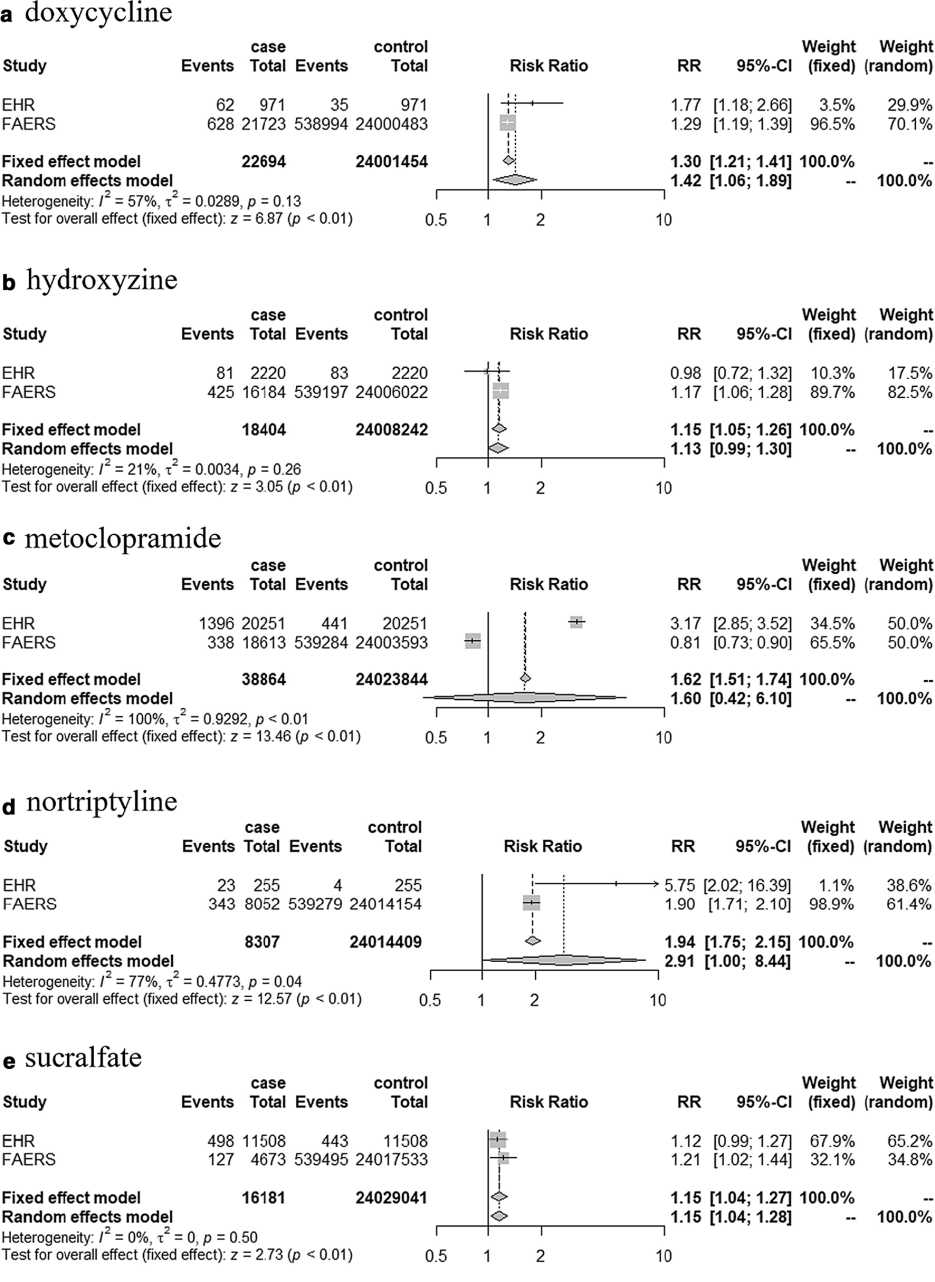
Risk ratio for ear disorders from KYUH-EHR and FAERS

A meta-analysis enables systematic and accurate conclusions to be made from the integrated results of individual studies. Through meta-analysis, we combined EHR and FAERS to increase statistical power with larger samples. Using the “metabin” function, the Risk Ratio (RR) was calculated by taking into account the number of events in case/control for each of the 9 drugs identified in EHR and FAERS. For the 9 drugs, the RR (and 95% CI) values in both models ([fixed effects model, random effects model]) were acetaminophen [0.94 (0.91–0.98), 1.14 (0.57–2.28)], cimetidine [0.79 (0.67–0.93), 0.82 (0.62–1.09)], digoxin [0.76 (0.69–0.83), 1.03 (0.50–2.14)], doxycycline [1.30 (1.21–1.41), 1.42 (1.06–1.89)], epinephrine [0.79 (0.72–0.87), 0.78 (0.50–1.23)], hydroxyzine [1.15 (1.05–1.26), 1.13 (0.99–1.30)], metoclopramide [1.62 (1.51–1.74), 1.60 (0.42–6.10)], nortriptyline [1.94 (1.75–2.15), 2.91 (1.00–8.44)] and sucralfate [1.15 (1.04–1.27), 1.15 (1.04–1.28)]. We generated a plot of the values, including both the random effects model and the fixed effects model in Fig. 6.

## Discussion

In this study, we have used a combination of diagnostics for ear disorders and PTA reports contained in the EHR to perform pharmacovigilance. The drugs that were detected in both EHR and FAERS were doxycycline, nortriptyline and sucralfate. The HR for nortriptyline was 8.06 in the EHR and the ROR was 1.94 in FAERS, whereas the value for sucralfate was about 1.22 in both the EHR and FAERS. These results showed that nortriptyline and sucralfate were potent drugs that caused ADRs of ear disorders. When the meta-analysis was conducted with a random effects model, the drugs with a significant RR were doxycycline (1.42), nortriptyline (2.91), and sucralfate (1.15). When using the fixed effects model, doxycycline (1.30), hydroxyzine (1.15), metoclopramide (1.62), nortriptyline (1.94), and sucralfate (1.15) were the drugs with a significant RR.

Through meta-analysis, we detected 5 drug-ADRs pairs for ear disorders, and classifications of each drugs are antibiotic, antidepressant, and etc. Of these, nortriptyline, which showed the highest signal in the meta-analysis, is an antidepressant. Previous studies have shown that antidepressants are effective in ameliorating tinnitus [33–35], but there has also been research concluding that there is no clear evidence to support the claim that antidepressants are effective in ameliorating tinnitus [36]. In addition, other side effects (e.g. dry mouth) are found, so their use as a treatment for tinnitus is limited [37]. Indeed, when we checked the signal of nortriptyline, it appears that it causes ear disorders, including tinnitus. Even if a drug works as a specific treatment, it should be examined using PDP for active surveillance for ADR detection. Therefore, we would detect the potential ADRs when the drugs are actually prescribed. The biggest advantage of PDP is that it can combine various medical data sources into a unified model, especially patient’s clinical information with laboratory results. It could provide evidence for pharmacovigilance researchers to detect drug induced ADRs.

To validate the performance of the presented data processing pipeline also for other diseases beyond ear conditions, we analyzed 2 additional System Organ Classes (SOCs) and conducted a meta-analysis. We considered commonly used measurements for analysis, then found the target blood and liver functions tests. The drugs were filtered using the drug selection algorithm described in the methods section. The blood tests used to detect neutropenia and thrombocytopenia were absolute neutrophil count (ANC) and platelet levels, respectively. In the case of neutropenia, the RR using two models ([Fixed Effect Model, Random Effect Model]) for metoclopramide was 1.58 (1.42–1.77) and 3.28 (0.54–20.00), and that of palonosetron was 4.76 (3.95–5.74) and 6.64 (2.52–17.46) (see Additional file 1: Table S1). For thrombocytopenia, chlorpheniramine [1.84 (1.60–2.12), 1.98 (0.88–4.44)], and palonosetron [5.25 (1.14–2.23), 3.81(0.59–24.64)] showed the signals (see Additional file 1: Table S2). In case of the hepatobiliary disorder, acute hepatitis, liver function tests such as aspartate aminotransferase (AST) and alanine aminotransferase (ALT) were selected for use in the meta-analysis. Metoclopramide [4.24 (3.81–4.73), 3.25 (1.62–6.53)], and nicardipine [8.21 (6.92–9.74), 9.28 (5.94–14.51)] showed signals of acute hepatitis (see Additional file 1: Table S3). We could find several novel drug-ADR pairs using the measurements in other SOCs through PDP.

There are some limitations to this study: 1) we analyzed EHR data of only one institution; 2) the EHR data contains fewer patients. First, in general, the use of only one hospital for analysis may make it difficult to demonstrate the validity of a signal. Therefore, we developed a PDP to combine EHR data and SRS data, and used additional information such as the PTA reports of EHR data for analysis. The problem of the small number of patients in EHRs may be overcome through meta-analysis with the FAERS result. Owing to the relatively small number, it was statistically analyzed with the results of sufficiently large numbers of data.

As the SRS data contains valuable and extensive information about the relationship between drugs and ADRs compared with other medical data sources, we will use Korean SRS data in future studies. We intend to expand the PDP with Korea Adverse Event Reporting System (KAERS) data, similar to FAERS. As KAERS also uses other types of terminology, the information should first be converted to a CDM and then the appropriate tables should be analyzed.

## Conclusions

EHR has well-structured time-dependent information and covariate measurements, but there are complexities in the identification and definition of ADRs, whereas FAERS contains clear definitions of ADRs, but does not include time-based information and inspection records [38]. We proposed a pharmacovigilance-specialized data process pipeline, PDP, that combines different real-world data sources for active surveillance of potential ADR signals. We were able to relate PTA reports in the EHR by using the PDP, and we focused on novel drugs that may induce ear disorders. For disorders of other system organ classes, we can also relate the other measurement or test results in the EHR. We found five drugs that could be related with potential ADR signals for ear disorders that were common to both data sources: doxycycline, hydroxyzine, metoclopramide, nortriptyline, and sucralfate. Currently, these drugs are not reported to cause ear disorders. If we expand our PDP further, we are confident that it will allow the detection of novel drugs that have signals for a wide range of ADRs, as shown in this study.

## Supplementary Information

**Additional file 1. Figure S1**. Risk ratio for Blood and Lymphatic system disorders from KYUH-EHR and FAERS (Neutropenia).** Figure S2**. Risk ratio for Blood and Lymphatic system disorders from KYUH-EHR and FAERS (Thrombocytopenia).** Figure S3**. Risk ratio for Hepatobiliary disorders from KYUH-EHR and FAERS (Acute hepatitis).

## Data Availability

The data used for this analysis included reports from FAERS collected between Q4 2012 and Q4 2018. FAERS is a publicly available database that contains information on adverse event and medication error reports submitted to FDA. The FAERS data utilized the code provided on https://github.com/ltscomputingllc/faersdbstats.

## Abbreviations

OMOP: Observation medical outcomes Partnership
CDM: Common data models
PDP: Pharmacovigilance data-processing pipeline
PV: Pharmacovigilance
ADRs: Adverse drug reactions
EHR: Electronic health records
SRS: Spontaneous reporting system
KYUH: Konyang university hospital
FDA: United states food and drug administration
FAERS: FDA adverse event reporting system
HR: Hazard ratio
ROR: Reporting odds ratio
US: United states
OHDSI: Observational health sciences and informatics
PTA: Pure tone audiometry
WHO: World health organization
CRF: Case report form
AEOLUS: Adverse event open learning through universal standardization
PTA6: Pure tone threshold average 6
PSM: Propensity score matching
RR: Risk ratio
SOCs: System organ classes
ANC: Absolute neutrophil count
KAERS: Korea adverse event reporting system
