# Supplementary material for: An OMOP-CDM based pharmacovigilance data-processing pipeline (PDP) providing active surveillance for ADR signal detection from real-world data sources
Source: BMC Med Inform Decis Mak. 2021 May 17;21:159. doi: 10.1186/s12911-021-01520-y (PMC8130307; doi:10.1186/s12911-021-01520-y)

S1. Risk ratio for Blood and Lymphatic system disorders from KYUH-EHR and FAERS (Neutropenia)

Blood and Lymphatic system disorders : Neutropenia

(a) metoclopramide

Study	case Events	case Total	control Events	control Total	RR	95%-CI	Weight (fixed)	Weight (random)
EHR	63	7803	302	305008	8.15	[6.22; 10.69]	3.7%	49.8%
FAERS	263	103986	221943	117003078	1.33	[1.18; 1.50]	96.3%	50.2%
**Fixed effect model**		**111789**		**117308086**	**1.58**	**[1.42; 1.77]**	**100.0%**	**--**
**Random effects model**					**3.28**	**[0.54; 20.00]**	**--**	**100.0%**

Heterogeneity: $I^2 = 99\%$, $\tau^2 = 1.6876$, $p < 0.01$
Test for overall effect (fixed effect): $z = 8.27$ ($p < 0.01$)

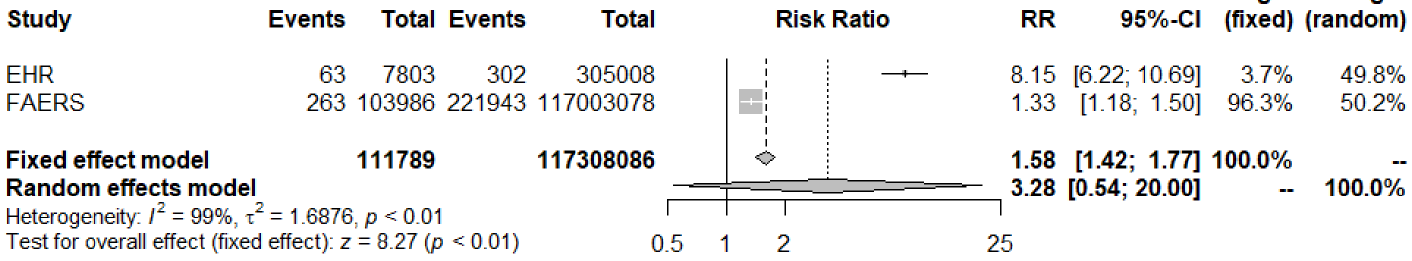

(b) palonosetron

Study	case Events	case Total	control Events	control Total	RR	95%-CI	Weight (fixed)	Weight (random)
EHR	23	1899	342	310912	11.01	[7.24; 16.75]	9.0%	48.2%
FAERS	87	11075	222119	117095989	4.14	[3.36; 5.11]	91.0%	51.8%
**Fixed effect model**		**12974**		**117406901**	**4.76**	**[3.95; 5.74]**	**100.0%**	**--**
**Random effects model**					**6.64**	**[2.52; 17.46]**	**--**	**100.0%**

Heterogeneity: $I^2 = 94\%$, $\tau^2 = 0.4592$, $p < 0.01$
Test for overall effect (fixed effect): $z = 16.37$ ($p < 0.01$)

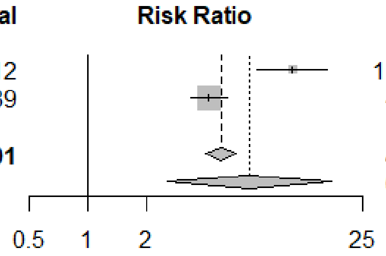

S2. Risk ratio for Blood and Lymphatic system disorders from KYUH-EHR and FAERS (Thrombocytopenia)

Blood and lymphatic system disorders : Thrombocytopenia

(a) chlorpheniramine

Study	case Events	case Total	control Events	control Total	RR	95%-CI	Weight (fixed)	Weight (random)
EHR	132	78354	299	234523	1.32	[1.08; 1.62]	68.0%	49.9%
FAERS	104	17639	233945	117077582	2.95	[2.44; 3.57]	32.0%	50.1%
**Fixed effect model**		**95993**		**117312105**	**1.84**	**[1.60; 2.12]**	**100.0%**	**--**
**Random effects model**					**1.98**	**[0.88; 4.44]**	**--**	**100.0%**

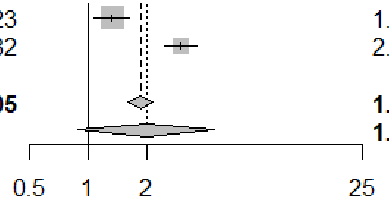

Heterogeneity: $I^2 = 97\%$, $\tau^2 = 0.3307$, $p < 0.01$
Test for overall effect (fixed effect): $z = 8.49$ ($p < 0.01$)

(b) palonosetron

Study	case Events	case Total	control Events	control Total	RR	95%-CI	Weight (fixed)	Weight (random)
EHR	38	17874	393	295003	1.60	[1.14; 2.23]	50.6%	49.7%
FAERS	197	10965	233852	117084256	9.00	[7.83; 10.33]	49.4%	50.3%
**Fixed effect model**		**28839**		**117379259**	**5.25**	**[4.61; 5.97]**	**100.0%**	**--**
**Random effects model**					**3.81**	**[0.59; 24.64]**	**--**	**100.0%**

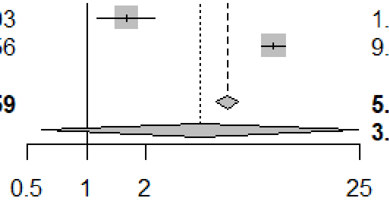

Heterogeneity: $I^2 = 99\%$, $\tau^2 = 1.7971$, $p < 0.01$
Test for overall effect (fixed effect): $z = 25.20$ ($p < 0.01$)

S3. Risk ratio for Hepatobiliary disorders from KYUH-EHR and FAERS (Acute hepatitis)

Hepatobiliary disorders : Acute hepatitis

(a) metoclopramide

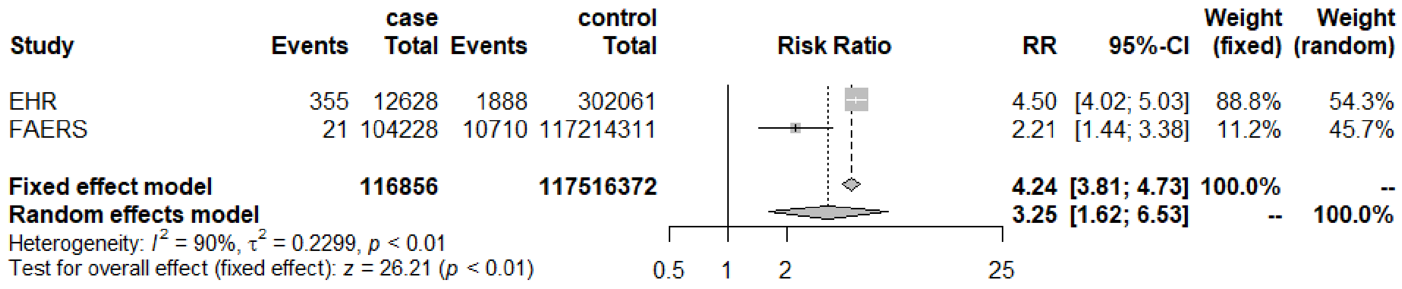

(b) nicardipine

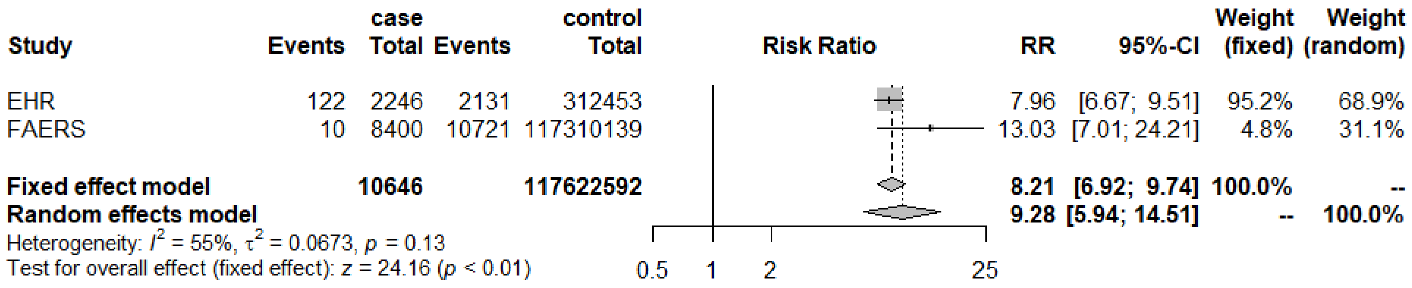

Supplement: Supplementary file 1 — Additional file 1. Figure S1. Risk ratio for Blood and Lymphatic system disorders from KYUH-EHR and FAERS (Neutropenia). Figure S2. Risk ratio for Blood and Lymphatic system disorders from KYUH-EHR and FAERS (Thrombocytopenia). Figure S3. Risk ratio for Hepatobiliary disorders from KYUH-EHR and FAERS (Acute hepatitis). [file 12911_2021_1520_MOESM1_ESM.pdf]
